# Molecular characterization of *Shigella* spp. isolates from a pediatric hospital in Southwestern Iran 

**Published:** 2017

**Authors:** Leili Shokoohizadeh, Gholam Abbas Kaydani, Alireza Ekrami

**Affiliations:** 1 *Infectious and Tropical Diseases Research Center* ***, ***Health *Research* Institute*, Ahvaz Jundishapur University of Medical Sciences, Ahvaz, Iran*; 2 *Department of Medical laboratory Sciences, Faculty of Para Medicine, Ahvaz Jundishapur University of Medical Sciences, Ahvaz, Iran *

**Keywords:** *Shigella*, Multi-Locus Sequence Typing, Iran

## Abstract

**Aim:**

In This study focused on the detection of dominant clones and genetic relationship of *Shigella* spp. isolated from children with diarrhea in the main pediatric hospital in Ahvaz by multi-locus sequence typing (MLST) technique.

**Background:**

Shigellosis is considered as one of the problematic bacterial infections for public health in the world. Khuzestan province in the Southwestern part of Iran is a known endemic area for infections due to *Shigella*. There are limited molecular epidemiological data for *Shigella* spp. in this area.

**Methods:**

A total of 50 *Shigella* spp. were isolated from January-June 2015 based on conventional microbiology and serology tests. The Sequence types (ST) of *Shigella* isolates which are characterized by *Enterobacterial* repetitive intergenic consensus (ERIC-PCR) were detected by MLST technique.

**Results:**

Among 50 *Shigella* isolates, a total of 31(62%), 16(32%) and 3 (6%) of *Shigella* isolates were identified as *S. flexneri*, *S.sonneii*, and *S.boydii*, respectively. Two different sequence types (ST152 and ST245) were identified in *Shigella* isolates. ST152 was detected in S*.sonnei* and ST245 in *S. flexneri* and *S. boydii* isolates.

**Conclusion:**

Based on MLST data, the stable and genetically linked *Shigella* clones are the cause of *Shigella* infections in children in Southwestern Iran.

## Introduction

*Shigella *is a member of the Enterobacteriaceae family which is the cause of shigellosis with diarrhea, fever and abdominal cramps in infected persons. *S. flexneri, S. dysenteriae, S. sonnei, S. boydii* are clinically important species of *Shigella* genus (1). Most cases of shigellosis are observed in children. *Shigella* is known as one of the main causes of death among children worldwide, as well as in Asia (2). 

Khuzestan province in the Southwestern part of Iran is a known endemic area for *Shigella* infections in children. Despite the importance of *Shigella*, there is no adequate information regarding molecular epidemiology studies on *Shigella* infections in this area. 

Molecular typing has been considered as a useful tool for tracing the sources of bacterial infections and genetic relationship of the bacteria in epidemiological studies (3, 4).

PCR-based typing methods, such as Enterobacterial repetitive intergenic consensus (ERIC-PCR) is a technique for producing fingerprint directly without treatment by endonucleases (5).

Multi-locus sequence typing (MLST) has been known as a tool for epidemiological studies to explore the clonal lineages and evolutionary pathways of bacteria. MLST method is one of the PCR-based typing methods. This technique delivers comparable data through internet among different areas of the world. Isolates of microbial species characterized by their unique allelic profiles using the DNA sequences of internal fragments of multiple housekeeping genes in this procedure (6, 7). The alleles at each of the loci define the allelic profile or sequence type (ST). 

Given the rare information of *Shigella* clones in Iran and also in Khuzestan, the aim of this study was to detect the genetic relatedness and sequence type of *Shigella* spp. isolated from children in Ahvaz (center of Khuzestan) by MLST which was characterized by ERIC-PCR. 

## Methods

**Identification of** ***Shigella*** **isolates**

The study included all *Shigella *strains isolated from all cases of shigellosis in patients who had been admitted to Abuzar Pediatric hospital in Ahvaz, from January to June 2015**.** *Shigella *isolates were detected at the genus and species levels during the study period from diarrheal stool samples based on the conventional microbiologic and serologic tests (8), using specific anti sera (Bahar afshan, Iran).

**ERIC-PCR**

Genomic DNAs were extracted from *Shigella* isolates by commercial DNA extraction kit (Sinaclon, Iran). ERIC-PCR was performed as described previously (9). Banding patterns of ERIC-PCR were analyzed using GelCompar software version VI. ERIC profiles were compared with Dice method and clustering was performed by Unweighted Pair Group Method with Arithmetic Mean [UPGMA] program.

**Multilocus Sequence Typing**

Further characterization of *Shigella* isolates was detected by MLST. The internal fragments of seven housekeeping genes (*adk*, *fumC*, *gyrB*, *icd*, *mdh*, *pur*A and *rec*A) were amplified using the specific primers and PCR conditions by referring to online MLST database: http://mlst.warwick.ac.uk/mlst/dbs/ which was introduced for MLST of *E. coli*. PCR products were purified and sequenced (Macrogen, Seoul, Korea). Allele’s number and STs of *Shigella *isolates were assigned based on the order in the online MLST database.

The ability of ERIC and MLST to differentiate between different isolates (discriminatory power) was calculated by the number of types and the index of discrimination or ID (http://biophp.org/stats/discriminatory_power/demo.p).

## Results

***Shigella*** **serogroups**

A total of 50 *Shigella* isolates were identified, of these, 31(62%), 16(32%) and 3 (6%) of Shigella isolates were identified as *S. flexneri*, *S.sonneii*, and* S.boydii, *respectively*.* *Shigella* isolates were isolated from 21(42%) female and 29(58%) male patients. Sixty –four (68%) patients were 2-8 years old and 16(32%) of them were under 2 years old. 

**ERIC-PCR**

**Figure 1:**
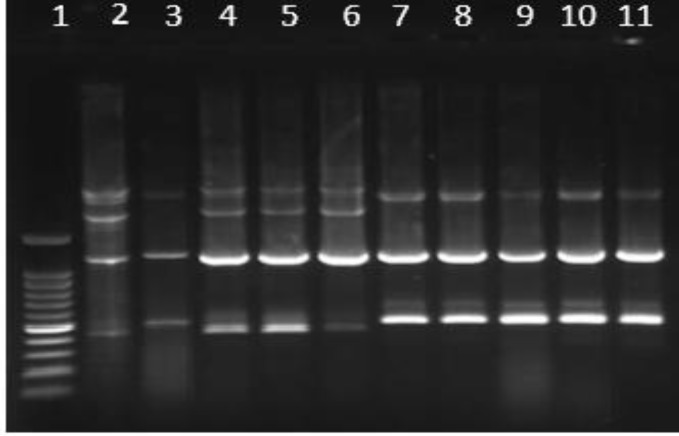
Gel electrophoresis image of ERIC-PCR of *Shigella* spp**.** Lanes: 1, 100 bp Plus DNA ladder; 2, *S.Sonnei*; 3, *S.boydii*; 4-6; S. *Sonnei*, 7-11, *S. flexneri*

**Figure 2:**
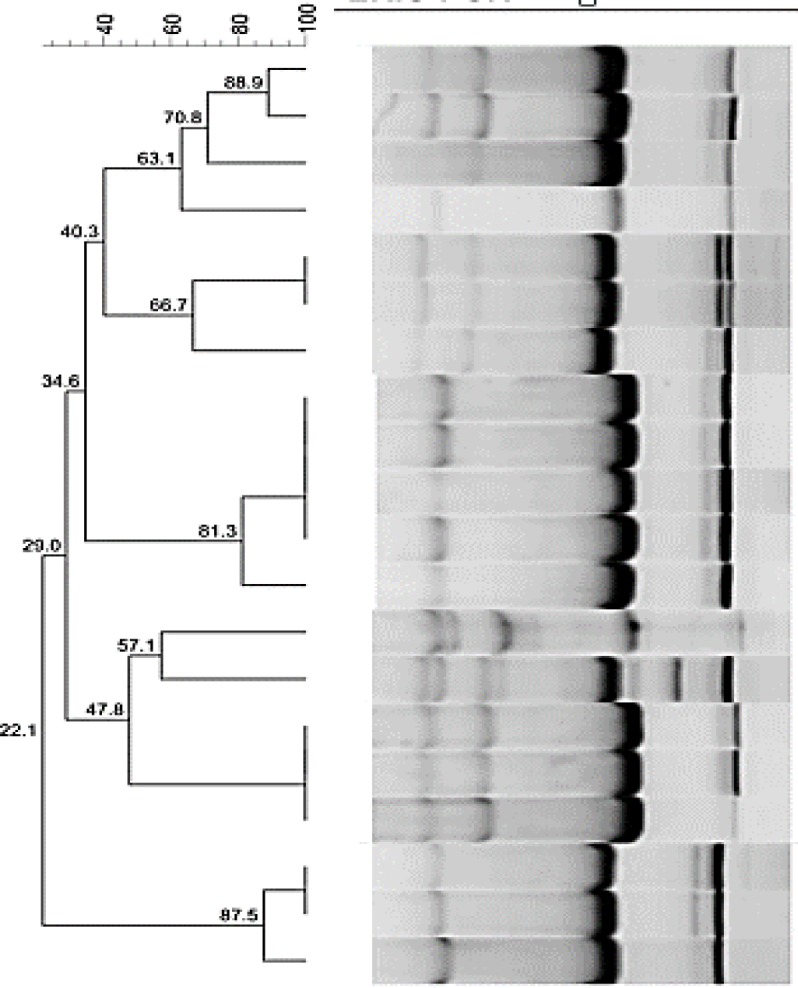
ERIC-PCR analysis of *Shigella* spp. Isolated from a pediatric hospital in Ahvaz. *Shigella* isolates were clustered and compared by UPGMA and Dice methods

ERIC-PCR analysis results revealed genetic diversity among *Shigella* isolates (Figure 1). ERIC results revealed eleven different types of ERIC, results reported previously (Figure 2). ERIC profiles of *S. flexneri*, *S. sonnei *and *S. boydii *were different from each other. Each species of *Shigella* exhibited high genetic relationship (9). The discriminatory power for ERIC-PCR was 0.91.

**Multi locus sequence typing**

 Based on MLST analysis, a total of two different sequence types were detected in this study including ST152 and ST245. ST152 was detected in *S. sonneii* strains and ST245 was found in *S. flexneri* and *S. boydii* strains. The allelic profiles of *adk*, *fumC*, *gyrB*, *icd*, *mdh*, *pur*A and *rec*A were 11, 63, 7, 1, 14, 7 and 7 in ST152 and 6, 61, 6, 11, 13, 3 and 50 in ST245. The calculated discriminatory power for MLST was 0.52.

## Discussion

In this study, *flexneri *was identified as the most common serogroup; however in recent years, studies from Tehran, Iran indicated *S. sonnei* as the main serogroup of *Shigella* isolates (10- 12). 

Previous finding had showed different ERIC profiles among *S*. *flexneri*, *S. sonnei and S. boydii *strains. Indeed, ERIC-PCR was able to divide S*. flexneri *and *S. sonnei and S. boydii *isolates into separate ERIC patterns (9). 

 All of the detected STs in our study have been previously reported in other studies throughout the world (10, 13, 15, 16). Our results showed two different STs (ST152 and ST245) among *Shigella *isolates using MLST. ST152 is known as the most frequent type, which was also reported from Iran and other countries (10, 13, 15, 16). In line with other studies, ST152 was detected only in *S*.*sonnei* strains, however in a study from China by Cao et al, both *S. flexneri *and *S. sonnei *species were included in ST152 (13). In an experiment performed only on MLST of *Shigella* in Tehran, Iran, five ST (145, 152, 241, 245, and 1502) were detected by Shahsavan et al. and they reported STs 152, 241 and 502 in *S.sonnei* isolates and ST245 in S*.flexneri *(10). In our study, ST245 was detected in *S. flexneri* and *S. boydii* strains. This ST is completely different from ST152, all allelic profile of seven housekeeping genes of Shigella. The ST245 has also been detected in *S. flexneri* strains in Asia Pacific, Africa, America, and Europe and also in *S. boydii* strains in China (10, 14, 15, 16). Detection of the same STs of *Shigella* in two different regions of Iran and circulating ST152 and ST245 in Iran and other countries indicates the stability and genetic linkage of *Shigella* clones. However, more data are required for the study of *Shigella* clones in different parts of Iran.

In comparison with other typing method (e.g. ERIC-PCR, rep-PCR, plasmid profiling), MLST is a technique which is highly unambiguous and portable, with moderate to high discriminatory power, high repeatability and reproducibility and moderate to high cost (17). In this study, ERIC-PCR showed higher discriminatory power compared to MLST; 0.91 versus 0.52. ERIC-PCR showed more diversity within ST of *Shigella*. MLST sometimes lacks the discriminatory power to discriminate bacterial strains, which limits its use in epidemiological studies. MLST method might not possess the sufficient discriminatory power to distinguish between recent epidemiological events in short episodes; indeed this method is more suitable for organisms with a clonal evolution and phylogenetic relationships between species of bacteria, so, it is ideal for global epidemiology (17, 18). Our results showed the molecular characteristics including clonal relationship, and genetic linkage of *Shigella* spp. isolates from southwestern Iran, which increase our understanding of molecular characteristics of *Shigella* spp. and contributes to the prevention and control of shigellosis in southwestern Iran.
